# The iBobbly Aboriginal and Torres Strait Islander app project: Study protocol for a randomised controlled trial

**DOI:** 10.1186/s13063-019-3262-2

**Published:** 2019-04-05

**Authors:** Fiona Shand, Andrew Mackinnon, Kathleen O’Moore, Rebecca Ridani, Bill Reda, Mel Hoy, Todd Heard, Luke Duffy, Marian Shanahan, Lisa Jackson Pulver, Helen Christensen

**Affiliations:** 1 Black Dog Institute, University of New South Wales, Hospital Road, Randwick, NSW 2031 Australia; 2 Australian National University, Canberra, Australia; 3 Alive & Kicking Goals!, Broome, Australia; 4 Hunter New England Local Health District, Wiyiliin ta CAMHS, Newcastle, NSW Australia; 5 School of Medicine and Public Health, University of Newcastle, Newcastle, Australia; 6 National Drug and Alcohol Research Centre, University of New South Wales, Sydney, Australia; 7 Western Sydney University, Sydney, Australia

**Keywords:** Suicide, Suicide Prevention, e-health, e-mental health, app, Indigenous, Aboriginal and Torres Strait Islander, iBobbly

## Abstract

**Background:**

Suicide amongst Australian Aboriginal and Torres Strait Islander communities occurs at twice the rate of the general population and, with significant barriers to treatment, help-seeking prior to a suicide attempt is low. This trial aims to test the effectiveness of an app (iBobbly) designed with Aboriginal and Torres Strait Islander people for reducing suicidal ideation.

**Methods/design:**

This is a two-arm randomised controlled trial that will compare iBobbly to a wait-list control condition. The trial aims to recruit Aboriginal and Torres Strait Islander participants aged 16 years and over to test iBobbly, which is a self-help app delivering content based on acceptance and commitment therapy. The primary outcome for the study is suicidal ideation, and secondary outcomes include depression, hopelessness, distress tolerance, perceived burdensomeness and thwarted belonging, and help-seeking intentions. Data will be collected for both groups at baseline, post-intervention (after 6 weeks of app use), and at 6 months post-baseline (with a final 12-month follow-up for the iBobbly group). Primary analysis will compare changes in suicidal ideation for the intervention condition relative to the wait-list control condition using mixed models. An examination of the cost-effectiveness of the intervention compared to the control condition will be conducted.

**Discussion:**

If effective, iBobbly could overcome many barriers to help-seeking amongst a group of people who are at increased risk of suicide. It may provide a low-cost, accessible intervention that can reach more people. This trial will add to a sparse literature on indigenous suicide prevention and will increase our knowledge about the effectiveness of e-health interventions for suicide prevention.

**Trial registration:**

Australian New Zealand Clinical Trials Registry, ACTRN12614000686606. Registered on 30 June 2014.

**Electronic supplementary material:**

The online version of this article (10.1186/s13063-019-3262-2) contains supplementary material, which is available to authorized users.

## Background

Suicide intervention research is rare in Indigenous communities. While suicide rates for the general population in Australia have been relatively stable over the last 20 years, they continue to increase for Australian Aboriginal and Torres Strait Islander communities [1]. Despite a raft of policies, programs, and funding announcements aimed at suicide prevention for Indigenous Australians, suicide rates amongst Aboriginal and Torres Strait Islander people are currently twice that of the general population [2]. Against a history of dispossession, oppression, and disadvantage, suicide and help-seeking amongst Aboriginal and Torres Strait Islander people is different to what we see in the general population. In addition to a raft of structural issues such as poverty, social disadvantage, and poor physical health, there is a mistrust of mainstream services founded on a history of racism and mistreatment, a lack of services available to those in regional and remote areas, and a sense of shame. These issues are substantial barriers to treatment. Current initiatives are not achieving the desired outcome, with few Aboriginal and Torres Strait Islander people (hereafter respectfully referred to as Indigenous Australians) seeking help for their distress [3, 4] and even fewer finding effective services. Efforts directed at providing evidence-based help to those who don’t currently seek it are likely to reduce suicide rates.

Within the general population, reductions in suicidal ideation can be achieved using several face-to-face therapies, including cognitive behaviour therapy (CBT) [5], mindfulness-based CBT (MBCT) [6], and dialectical behaviour therapy (DBT) [7]. A recent iteration of CBT, acceptance and commitment therapy (ACT), has demonstrated effect sizes similar to those of traditional CBT, and superior effects to control conditions for a range of mental and physical health disorders [8]. ACT has demonstrated efficacy as a self-help intervention for chronic pain [9] and tinnitus [10] and as a prevention intervention for depression and anxiety symptoms [11]. An acceptance and emotional regulation-based intervention reduced self-harm in people with borderline personality disorder (BPD) [12]. Limited evidence shows that where ACT is being used in Indigenous communities it is acceptable and culturally aligned [13].

Web-based and self-help interventions have been found to reduce depression, anxiety, and suicide ideation, and may offer a solution to problems of service access and implementation [14–16]. In particular, apps downloaded to phones or mobile tablets then become available at all times, even without internet connection. Suicide prevention interventions offered via an app may have advantages by allowing people to seek help anonymously, compensating for a limited workforce, offering around-the-clock availability, and overcoming the stigma or shame barrier [3, 17]. An app is able to deliver interventions to a large number of individuals at a relatively low cost [18], and can be designed as an indicated preventively oriented intervention rather than as a response to crisis situations. When co-designed with young Aboriginal and Torres Strait Islander people, the chances of the intervention being unacceptable for cultural or age-related reasons can be expected to be much lower.

iBobbly is a self-help app developed to reduce suicidal ideation and plans. The app includes trans-diagnostic content from the most recent evolutions of cognitive behavioural therapy: ACT [19, 20], MBCT [6], and DBT [21]. The app was developed in consultation with Indigenous Australians and uses an ACT framework of values-based action, de-fusion from unhelpful thoughts, and acceptance of difficult emotions, drawing on values embedded within Indigenous culture. It was designed to be engaging and interactive whilst delivering an evidence-based therapy. iBobbly is designed to be accessible for people with lower literacy skills through the use of images, animations, and voice-overs created by Indigenous artists, graphic designers and speakers, and minimal use of text. Once downloaded the app also does not require the internet for ongoing use.

A pilot RCT of a prototype of the iBobbly app (version 1) was conducted in the Kimberley region of Western Australia between September 2013 and March 2015, involving 61 Indigenous participants aged 18–35 years. Participants were randomised to receive either the app, which delivered acceptance-based therapy over 6 weeks, or were waitlisted for 6 weeks and then received the app for the following 6 weeks. While the between-group changes in suicidal ideation from pre-intervention to post-intervention were not statistically significant, the pre–post intervention change for the iBobbly arm was statistically significant, showing a mean decline of 0.84 (95% CI 0.14–1.53) on the Depressive Symptom Inventory—Suicidality subscale [22]. This corresponds to a moderate effect size of 0.39 (Cohen’s d) and, in a preventive context, justifies further research using the app. In addition, participants in the iBobbly group showed substantial and statistically significant reductions in psychological distress compared with those in the waitlist. Change in impulsivity as measured in the trial was nonsignificant and negligible in size [23].

The current study differs from the pilot study in several ways. The major changes from the pilot study are the inclusion of several additional hypotheses (outlined below) and the inclusion of a cost-effectiveness analysis. This has resulted in additional outcome measures, including more comprehensive measures of suicidality, help-seeking, distress tolerance, interpersonal needs, service use, and quality of life. iBobbly itself is now a substantially different intervention, with major revisions based on feedback from the first pilot and from national consultations with a range of Aboriginal and Torres Strait Islander groups and communities. The study is recruiting a larger sample size from an additional five regions, representing a broader cross-section of Aboriginal and Torres Strait Islander communities. The lower age limit for participants has been reduced from 18 to 16. Finally, longer between-group (6 months) and intervention within-group (12 months) follow-up periods are included.

## Aims

### Primary aim

The primary aim of the trial is to test the effectiveness of the iBobbly app (version 2) compared to a waitlist control condition in reducing levels of suicidal ideation immediately post-intervention (6 weeks).

### Secondary aims

The secondary aims are as follows:

- ▪ To test the effectiveness of the iBobbly app compared to a waitlist control condition in reducing levels of suicidal ideation 6 months post-baseline.
- ▪ To test the effectiveness of the iBobbly app compared with the control condition for reducing symptoms of depression, hopelessness, perceived burdensomeness, and thwarted belongingness and for increasing quality of life, help-seeking intentions, and healthcare utilisation immediately post-intervention (6 weeks), and 6 months post-intervention.
- ▪ To examine the 12-month outcomes for participants randomised to the iBobbly group.

### Subsidiary aims

Subsidiary aims include to examine predictors of outcomes and non-response to the intervention, adherence to the app, acceptability of the intervention, trial dropout, and the cost-effectiveness of the intervention compared to the wait list control group.

## Trial hypotheses

In accordance with the above aims, the project is driven by several hypotheses:

- ▪ The iBobbly intervention will result in a reduction of suicidal ideation as measured by the Suicidal Ideation Attributes Scale (SIDAS) relative to the wait list control condition post-intervention (six weeks). The SIDAS is the primary outcome measure.
- ▪ The difference in suicidal ideation (measured by the SIDAS) between intervention and wait list control will be maintained at six months.
- ▪ For participants in the intervention group, reductions in suicidality will be maintained at the 12-month follow-up.
- ▪ The intervention will result in a reduction of reported suicide intent and plans as measured by the Quick Inventory of Depressive Symptomatology (QIDAS) relative to the control group at six weeks post-baseline and at six months.
- ▪ Relative to the wait list control condition, the intervention will lower symptoms of depression at six weeks post- baseline and six months.
- ▪ An increase in quality of life and self-reported help-seeking intentions is expected from baseline to six-month follow-up following use of the app.
- ▪ A reduction in suicidality (as measured by suicidal ideation, suicide plans and capacity to cope with suicide thoughts) will be associated with improvement in depression, improvement in burdensomeness and belongingness, improvement in distress tolerance, and greater program adherence.
- ▪ The cost effectiveness analysis will result in a favourable incremental cost effectiveness ratio for the intervention group relative to the control wait list with an improvement in quality of life, with higher costs associated with use of iBobbly app and treatment seeking, but lower use of crisis health care and social services (such as presentations to emergency department and inpatient units).

## Methods/design

A two-arm randomised controlled trial (RCT) will be conducted (one active intervention/one 6-month wait list control) using three measurement occasions: baseline, post-intervention (after completing the program at week 6), and follow-up at 6 months after baseline assessment. After community consultation, the waitlist control condition was selected as the only acceptable one to the Aboriginal and Torres Strait Islander communities involved in the trial. A SPIRIT checklist (Additional file 1) was completed to address all necessary items in the trial protocol.

### Community consultation

Following consultation with Aboriginal community members during research design it was decided that to ensure ethical standards and maintain appropriate duty of care, all participants would be provided with information about crisis services and offered referral to clinical services if needed. As such the study examines the impact of iBobbly compared to usual care in the waitlist group. The age range for recruitment was initially 16–35 years; however, following commencement of recruitment, feedback from research sites indicated that many community members over the age of 35 wished to take part and would likely benefit from the app; so to maintain positive relationships with community the upper age limit was removed. The initial follow-up period was to be 24 months but given difficulty with recruitment and feedback regarding burden of numerous follow-up questionnaires the 24-month follow-up was removed with the 12-month remaining as the final follow-up point for the iBobbly group.

### Recruitment

Community-dwelling Indigenous individuals will be recruited to this study via advertisements on social networking sites, Indigenous media, community events, and through established relationships with Aboriginal community health services.

Participants will be recruited from six regions across four Australian jurisdictions: Broome (Western Australia); Darwin (Northern Territory); Darling Downs (Queensland); and Hunter New England, La Perouse, and the Murrumbidgee region (New South Wales).

Eligibility to take part in the trial is determined in a stepwise screening process. To be eligible, participants must:

- ▪ Be at least 16 years of age
- ▪ Be of Aboriginal or Torres Strait Islander descent
- ▪ Be a resident of Australia and reside within an ethics-approved region (listed above)
- ▪ Have the ability to navigate a basic app
- ▪ Not be actively suicidal
- ▪ If diagnosed with a psychotic disorder such as schizophrenia not be currently experiencing symptoms
- ▪ Be willing to contact the Suicide Call Back Service (SCBS) if needed
- ▪ Be able to understand spoken English

### Randomisation

Randomisation of participants will occur with equal probability for both groups. Participants will be stratified based on gender (male/female) and severity of suicidal thoughts calculated from the SIDAS [24], using a cut-off score of 21 (subthreshold/high risk). Randomisation will be carried out at the Black Dog Institute by the trial manager using automated randomisation software for block randomisation (four per block). Participants were aware of allocation as they were provided the tablet with the app if randomised to the iBobbly group.

### Procedure

**Fig. 1:**
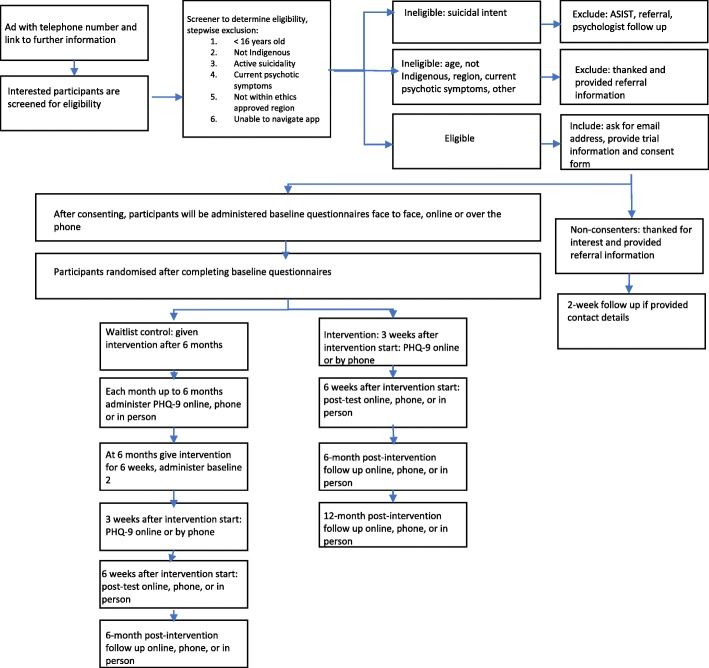
Participant flow through the trial

Upon recruitment, participants will be screened over the telephone or face-to-face using brief questionnaires to establish eligibility as per the criteria above. Participants will be excluded from the trial if they present with active suicidality at the time of recruitment, as indicated by a current plan to harm themselves or reporting having taken steps to put a suicide plan into place. These respondents will receive appropriate referral information to health services and crisis lines and will be supported to seek help (Fig. 1). Participants excluded on the basis of a diagnosis of schizophrenia or another psychotic illness will also be provided with referral information. To accommodate participants with varying literacy skills, individuals who meet the inclusion criteria will be given the option to complete baseline and follow up measures face-to-face or through a telephone interview with a site coordinator or the trial manager, or online.

Participants eligible to take part in the study will be given or emailed the Participant Information Statement, consent form and safety agreement by the site coordinator. Participants will also be asked to complete the participant contact form for us to be able to contact the participant at each follow-up point.

All participants will be administered the baseline measures, with those randomised to the intervention group given a mobile tablet device loaded with the app and provided with basic training on how to use it, as well as assistance with setting up their personal identification number. Participants in the wait list control condition will be given the mobile tablet and app 6 months after the intervention group. Active participants will be contacted every 3 weeks until the end of the intervention period, and waitlist participants every 4 weeks, via email or telephone for a wellbeing check. Participants in each group will be provided with information about suicide prevention services and mental health services. This involves direct access to suicide prevention crisis support, and referral to medical providers, if sought or if flagged as at-risk during wellbeing checks. In this instance, an Indigenous psychologist or counsellor will contact the participant and provide triage to local services or engage emergency services where required. Following community consultation it was decided that participant retention be supported through regular contact with the study team, and through a $20 gift card reimbursement for completing each assessment. All participants were free to withdraw from the study at any time.

### Intervention

iBobbly is a trans-diagnostic app-based self-help program aimed at reducing the frequency and intensity of suicidal ideation. As previously mentioned, the app uses culturally adapted content from ACT, MBCT, and DBT. It consists of three modules that focus on 1) dealing with unhelpful, distressing thoughts, including suicidal thoughts, 2) regulating intense emotions, and 3) values-based goal setting and action planning.

### Assessments

**Fig. 2:**
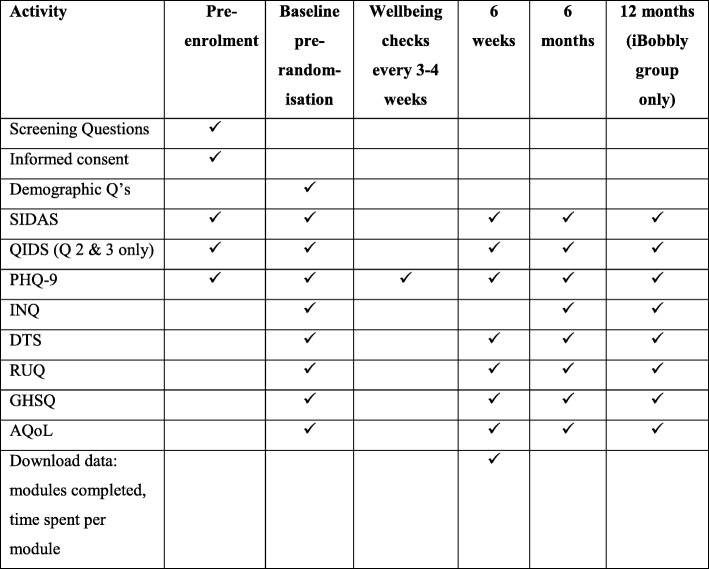
Phases of the trial and data collection points

Figure 2 presents the scales that will be administered at the various measurement occasions in the trial. It is estimated that it will take participants 20–30 min to complete each baseline, 6-week, 6-month, and 12-month questionnaires ranged depending on fluency with English. Where possible, scales adapted for or widely used among Aboriginal and Torres Strait Islander people were chosen. Study questionnaires are available by contacting the lead author. Where paper and pen questionnaires are used, data entry is completed either by site coordinators in each region via a secure online portal, or centrally at the Black Dog Institute. Random checks of 25% of questionnaires will be completed to validate the accuracy of data entry against paper questionnaires. Data cleaning will be completed at the end of the data collection period. Participant data are password protected and stored on secure University of New South Wales servers in de-identified form. Paper forms are kept in locked filing cabinets, with electronic transfer occurring via secure web services. All documents will be stored for 5 years after date of publication.

#### Demographic variables

The following demographic variables will be ascertained: age, gender, Aboriginal or Torres Strait Islander status, usual form of accommodation (own residence, parent, boarding, shelter, no fixed address/homeless, other), employment status (full-time, part-time, part-time and looking for full-time work, unemployed/looking for work, not in labour force), usual occupation (free text), educational attainment (some primary, all primary, some secondary, 4 years secondary, 6 years secondary), post-secondary or tertiary schooling (none, trade/apprenticeship, other certificate, diploma, bachelor or higher degree), and relationship status (single, de facto, separated, married, divorced, widowed).

#### Suicidality

The Suicidal Ideation Attributes Scale (SIDAS) [24] is a brief measure of severity of suicidal ideation. The five-item scale assesses frequency (item 1), controllability (item 2), closeness to attempt (item 3), distress (item 4), and interference with daily activities (item 5) over the past month. Respondents answer on an 11-point scale (ranging from 0 to 10) with total scores ranging from 0 to 50. Higher scores indicate greater suicidal ideation. Item 2 (controllability) is reverse scored. Those who endorse a frequency of 0 for the first question skip the remaining items and are given a score of 10 (full control) for item 2 (controllability). The SIDAS is suitable for online use. The scale has demonstrated high internal consistency with Cronbach’s alpha of 0.91, and good convergent validity. In an online survey of community-based Australian adults (*n* = 1352) [24], a cut-off of 1 had sensitivity of 85.5% for suicide plans and 84.0% for suicide attempts (with 67.1% and 63.6% specificity, respectively). However, high specificity is needed to identify individuals most likely to engage in suicidal behaviour. At a cut-off score of 21, the SIDAS has 95.8% specificity for presence of plan in the past year and 94.9% specificity for presence of preparation for or making a suicide attempt in the past year. The suicidal intent and suicidal plan questions of the Quick Inventory of Depressive Symptomatology (QIDS) will be used to assess suicide plans.

#### Help-seeking

The General Help-Seeking Questionnaire (GHSQ) is a brief measure of a person’s intentions to seek help for emotional or personal problems and suicidal thoughts [25]. The measure consists of two questions, the first asking the likelihood of seeking help for an emotional or personal problem and the second asking the likelihood of seeking help for suicidal thoughts. The two questions list 11 possible sources someone might ask for help, e.g. their friend, doctor, etc., and includes the statement “I would not seek help from anyone” as a source. Each source is rated on a seven-point scale from 1 (extremely unlikely) to 7 (extremely likely). The scale demonstrates good reliability (α = 0.83 for the suicidal ideation question and α = 0.70 for the emotional/personal problems question) and validity amongst a sample of Australian high school students [25].

The Resource Use Questionnaire (RUQ) is a modified version of the Client Socio-Demographic and Service Receipt Inventory—European Version (CSRI) [26]. The language in the questionnaire has been simplified to suit Indigenous Australians where English may not be their first language. The RUQ captures service use measures, including contact with mental and primary health care staff and medication usage. A comparison of GP records with data from the CSRI found no significant difference in the average number of GP visits recorded on GP case records (mean = 3.03; sd = 3.49) and on the RUQ (mean = 2.99; sd = 3.67), and the agreement between the two sources was high (concordance correlation coefficient 0.756) [27]. The measure was reviewed by Indigenous staff for appropriateness for economic evaluation within the Aboriginal community.

#### Depression symptoms

The Patient Health Questionnaire (PHQ-9) is the depression scale of the PHQ, and consists of nine items, each scored 0 (not at all) to 3 (nearly every day). Participants are asked to rate how problematic the items have been for them over the past 2 weeks, yielding an overall score ranging from 0 to 27. The scores are grouped into five categories: 0–4 representing no/minimal depression; 5–9 representing mild depression, 10–14 moderate, 15–19 moderately severe, and 20–27 severe depression. The PHQ-9 has demonstrated good internal validity in participants attending primary care clinics (α = 0.89, *n* = 6000) and obstetric patients (α = 0.86, *n* = 3000). It has an acceptable test–retest reliability of kappa = 0.84 [27]. An adaptation of PHQ-9 for Indigenous Australian communities was utilised in this study [28], and a validation study is currently underway [29].

#### Distress tolerance

The 15 items on the Distress Tolerance Scale (DTS) measure a person’s ability to experience and tolerate negative psychological states [30]. Scored on a five-point scale from strongly agree (1) to strongly disagree (5), a higher overall score on the scale indicates a higher perceived capacity to tolerance distress (range = 15 to 75). The DTS has been found to be a valid measure of distress tolerance in a non-clinical university student sample [30] and a wider community sample of smokers [31]. Cronbach’s alpha for the overall scale was found to be 0.91 [31].

#### Interpersonal needs

The Interpersonal Needs Questionnaire (INQ) is comprised of 25 items that measure thwarted belongingness and perceived burdensomeness [32]. The respondent reports how they have been feeling recently on ten items that measure belongingness and 15 items that measure burdensomeness. The items are rated on a seven-point scale from 1 (not true at all) to 7 (very true). Higher overall scores indicate higher levels of thwarted belongingness and perceived burdensomeness (range = 18 to 126). The INQ has been found to be a reliable and valid measure of belongingness and burdensomeness in younger and older adults in clinical and non-clinical samples [32]. Comparable internal consistency coefficients were found for the belongingness (α = 0.85) and the perceived burdensomeness items (α = 0.89) and construct validity data were encouraging (e.g., thwarted belongingness, more so than perceived burdensomeness, correlates with a theoretically related interpersonal construct, i.e., loneliness) [32].

#### Assessment of quality of life (AQoL-8D)

The AQoL-8D will be used to assess health related quality of life [33]. It is commonly used to calculate utility scores before and after intervention for use in economic evaluation studies. The AQoL-8D can be completed in 5 minutes and is comprised of 35 items from which eight dimensions and two ‘super-dimensions’ are derived. The 35 items may be reduced to a single utility score using the AQoL-8D algorithm. AQoL-8D score ranges from full health (1.00) to health states worse than death (− 0.04) [34]. In addition, the algorithm produces an index number for each of the eight dimensions and for the two ‘superdimensions’, ‘Physical super-dimension’ (PSD; independent living, pain, senses) and ‘Mental super-dimension’ (MSD; mental health, happiness, coping, relationships, self-worth). The AQoL-8D has been shown to have good criterion validity, with Cronbach’s Alpha of 0.97.

### Statistical analysis

#### Analysis plan

Primary analyses will be undertaken on an intent-to-treat basis, including all participants as randomised, regardless of treatment actually received or withdrawal from the study. Likelihood based methods (mixed-model repeated measures [MMRM]) methods will be used to analyse change in the primary outcome measure (SIDAS). An a priori planned comparison of change from baseline to the post-intervention endpoint (6 weeks) will be used to test the primary hypothesis. An unstructured variance–covariance matrix will be used to accommodate relationships between observations at different occasions. The stratification factor of gender will be included in these models and variables found to be substantially imbalanced (equating to a moderate effect size) between groups post-randomisation will be tentatively included but not retained to evaluate the robustness of conclusions reached. Similar analyses of continuous/scaled secondary measures will assess differential change due to intervention arm. Mathematical transformation or categorisation of raw scores into ordered or dichotomous classes may be undertaken to meet distributional assumptions and address any violation of assumptions attributable to outliers. MMRM assumes data are missing at random [35, 36]. As this assumption cannot be substantiated from observed data, sensitivity analyses will be undertaken to evaluate effects of missing data and the robustness of the trial’s conclusions. As appropriate, these will be based on plausible models of missingness informed, as much as possible, by the experience of those working closely with participants.

For dichotomous outcomes such as caseness, a comparable generalized mixed modelling approach will be used. Number needed to treat [37] to avoid high risk of suicidal behaviour will be derived from the SIDAS using a cut-off score of 21 [24]. Number needed to treat to avoid moderate depression will be derived from the PHQ-9 using a cut-off score of 10 [38]. Separate analyses will incorporate 6-month follow up data. At this stage it will be possible to estimate post-intervention change in the active group and also to estimate change in the wait-list group after use of the app; however, comparison with an untreated sample will not be possible. All tests of treatment effects will be conducted using a two-sided alpha level of 0.05 and 95% confidence intervals.

#### Cost effectiveness analyses

Relevant costs will be applied to identified resource use during the trial, including the costs of the intervention, other health services utilisation (including use of ambulance, visits to hospital, medications, and physician contacts) and out-of-pocket expenses (cost to the individual for mental health and social services care). The primary outcome for the CEA will be quality of life (QALY) as measured using AQoL-8D. Change in suicide ideation (SIDAS) will be used as a secondary outcome. An incremental cost-effectiveness ratio (ICER) will be calculated for both the QALY outcome and the suicidal ideation outcome. Confidence intervals will be estimated around the ICER using non-parametric bootstrapping, and a cost-effectiveness acceptability curve will be estimated.

#### Sample size and power calculations

Power to detect change in ideation is based on the expected effect of the intervention at the 6-week time point on the primary outcome measure (SIDAS) after completion of the intervention as delivered by the app (6 weeks post baseline). Expected effects were based on the findings from a recent Dutch trial using a modified CBT website in the general population [39] which reported an effect size of 0.28 (Cohen’s d). A smaller effect than this on suicidal ideation would mean the app would be of limited interest as an intervention. To be able to detect this effect size, assuming a correlation between baseline and post-intervention measures of 0.5, including an expected drop-out rate of 30%, 289 participants, yielding complete data from 202 people, will be needed in each condition (with alpha = 0.05 and power of 0.80). This will also result in good precision, yielding confidence intervals of width approximately ± 0.20 for the effect size. US data show that 33.4% of adolescents with suicidal ideation also report plans [40]. Assuming this rate of plan reporting in the control condition, a reduction of plans under the active intervention to 20.5% (relative risk 0.61) would be required to maintain 80% power using Fisher’s exact test. While the study is not explicitly powered to detect this outcome, it is plausible that reduction in rates of plan-making may be detected with the target sample size. It may be feasible to determine when plans were made, allowing the testing of hypotheses regarding plans using more clinically informative methods such as survival analysis.

#### Data monitoring and governance

A Data Safety Monitoring Board (DSMB) has been established in accordance with the Australian National Health and Medical Research Council Guidelines. The DSMB is independent of the funders, has no competing interests, and its members are not study investigators. The DSMB is comprised of three senior academics experienced in conducting randomised controlled trials in suicide prevention and mental health. One member is also a biostatistician. The DSMB will receive 6-month data reports and will be notified of adverse events, as required by the human research ethics committee (HREC). Their findings on adverse events will be reported to the HREC. The DSMB has the authority to stop the trial if interim analyses indicate that sufficient sample size has been achieved, or there is sufficient evidence that the intervention is causing harm. Site coordinators will report adverse events to the trial manager within 72 h of notification of the event. Police and postvention services in each site have been asked to advise the trial manager of any deaths by suicide within the region. The investigator team acts as the steering committee. The trial manager reports to the principal investigator, and the principal investigator is responsible for liaising with the DSMB and the HREC. Site coordinators report to the trial manager. Protocol amendments will be reported to the investigators and submitted to all ethics committees for their approval prior to implementation.

### Dissemination of findings

Authorship on peer-reviewed manuscripts will be offered to those who have made a significant contribution to the conception or design of the project or the acquisition, analysis, or interpretation of data for the work; and/or drafted the work or reviewed it critically for important intellectual content. There are no publication restrictions for this research. Dissemination of results will be via traditional channels of journal publications and scientific conferences. In addition, there is a clear obligation under research guidelines for working with Aboriginal and Torres Strait Islander communities to report results back to participants and involved others. This will occur through the production and dissemination of plain language reports and diagrams and presentations to communities. Budget will be sought for production of an explainer video. Only aggregated results will be reported with a minimum cell size of 5, in order to protect participants’ confidentiality.

## Discussion

Intervention research in suicide prevention is rare and in Indigenous communities it is all but non-existent [41, 42]. Efforts to reduce suicide amongst Indigenous Australians have thus far not achieved their outcomes, so this research is critical if our knowledge of Indigenous suicide prevention is to improve [3, 4]. This project has the potential to reach young Indigenous people at risk of suicide who are unable or unwilling to undergo face-to-face treatment with health professionals. The research will have both theoretical and practical international significance. If this app is found to be effective, it will be immediately deployable for use by consumers individually and in conjunction with suicide crisis agencies in the Australian community (and the English speaking international community, including those with low literacy skills). The program could also prove useful as an adjunct to community health services, general practice, workplaces, and schools, particularly where access to mental health services is limited.

At a population level, the prevalence of suicidal ideation, attempts and deaths may be lowered. The potential effects on attempts and completions are difficult to model as no effect measures are available for these outcomes at this stage, given there are low base rates. Indigenous Australians have higher rates of smart phone ownership compared to non-Indigenous Australians and have been shown to access social media sites more frequently [43]. Therefore, smart phone and mobile tablet technology enables large scale deployment. If the effectiveness of iBobbly is established, the intervention will be offered broadly, and its reach and impact measured systematically. If effective, it will establish new models for the delivery of suicide prevention in Australia by combining an effective intervention within the safety net of a crisis intervention or general practitioner service. Finally, the study represents the opportunity to undertake highest quality intervention research in Indigenous communities with the engagement and support of Indigenous leaders. It thus represents an opportunity to raise the benchmark of mental health research in this sector into the future.

## Trial status

The trial was open for enrolment of participants between 6 September 2016 and 31 April 2018. The current protocol article was submitted to *Trials* before all participants were enrolled and before breaking the randomisation code or any analysis of data.

## Additional file

Additional file 1: SPIRIT 2013 checklist: Recommended items to address in a clinical trial protocol and related documents. (DOC 120 kb)

## Abbreviations

ACT: Acceptance and commitment therapy
AQoL-8D: Assessment of Quality of Life
BPD: Borderline personality disorder
CBT: Cognitive behaviour therapy
DBT: Dialectical behaviour therapy
DTS: Distress Tolerance Scale
GHSQ: General Help-Seeking Questionnaire
ICER: incremental cost effectiveness ratio
INQ: Interpersonal Needs Questionnaire
MCBT: Mindfulness-based cognitive behaviour therapy
MMRM: Mixed-model repeated measures
PHQ-9: Patient Health Questionnaire
QIDAS: Quick Inventory of Depressive Symptomatology
RCT: Randomised controlled trial
RUQ: Resource Use Questionnaire
SCBS: Suicide Call Back Service
SIDAS: Suicidal Ideation Attributes Scale
